# Exploratory Factor Analysis of Rainbow Trout Serum Chemistry Variables

**DOI:** 10.3390/ijerph18041537

**Published:** 2021-02-05

**Authors:** Maurizio Manera

**Affiliations:** Faculty of Biosciences, Food and Environmental Technologies, University of Teramo, St. R. Balzarini 1, 64100 Teramo, Italy; mmanera@unite.it; Tel.: +39-0861-266980

**Keywords:** exploratory multivariate analysis, fish wellness, fish physiology, fish biochemistry

## Abstract

Clinical chemistry offers a valuable, affordable, moderately invasive, and nondisruptive way to assess animal physiological status and wellness within defined ranges and is widely used as a diagnostic clinical tool. Because of physiological differences between mammals, clinical correlates of blood chemistry variables are not known in detail in fish, in which tissue/organ function tests are inferred from mammal-derived clinical chemistry data. The aim of the present study was to apply exploratory factor analysis on a serum chemistry dataset from clinically healthy, reared rainbow trout *Oncorhynchus* *mykiss* (Walbaum, 1792) to select the most correlated variables and to test for possible underlying factors explaining the observed correlations as possible physiological status estimates in trout. The obtained factors were tested for correlation with hepatosomatic and splenosomatic indexes. Thirteen highly correlated variables were selected out of 18 original serum chemistry variables, and three underlying factors (Factors 1, 2, and 3) were identified that explained the observed correlations among variables. Moreover, Factor 1 correlated negatively with the hepatosomatic index and Factors 2 and 3 negatively with the splenosomatic index. The obtained factors were tentatively associated with: protein (liver) metabolism (Factor 1), cell turnover (Factor 2), and lipid (muscle) metabolism (Factor 3).

## 1. Introduction

Clinical chemistry offers a valuable, affordable, moderately invasive, and nondisruptive way to assess animal physiological status and wellness within defined ranges and is widely used as a diagnostic clinical tool [1]. With regard to fish, problems arise regarding the definition of normal ranges because of the possible influence of many internal and external environmental factors [2,3,4]. Moreover, and as a result of physiological differences between fish and mammals, from which clinical chemistry methods have been developed, clinical correlates of blood chemistry variables are not known in detail. For instance, and generally speaking, fish are ammonotelic animals relying on gills for nitrogenous waste clearance, whereas mammals are ureotelic animals, excreting nitrogenous waste mainly in the form of urea through the kidney [5,6,7]. In fish, kidney functionality relies on evolutionary consideration related to the environment in which each single species has evolved [8,9]. Therefore, blood chemistry variables in fish may account for different and not completely understood morphofunctional and pathophysiological features and may correlate differently with each other. As a clinical chemistry routine in human and veterinary medicine, more than one variable is used to assess tissue/organ functionality. These markers of tissue/organ functionality are selected based on possible physiological and/or pathophysiological correlations among the chosen variables. As a consequence, there are, e.g., liver function and kidney function tests [1,10,11]. To date, this is not the case in fish, where tissue/organ function tests are inferred from mammal-derived clinical chemistry data, therefore deserving further research. Fish farming has dramatically increased over recent years, with fish being widely used for human and animal nutrition, in environmental and biomedical research [12,13,14]. As a result, there is an urgent, mandatory need to define fish physiological status and wellness criteria to be applied in accordance with regulations in force worldwide [12].

The aim of the present study was to apply exploratory factor analysis on a serum chemistry (normal ranges) dataset previously obtained in clinically healthy, reared rainbow trout *Oncorhynchus mykiss* (Walbaum, 1792) in order to select the most correlated variables and to test for possible underlying factors explaining the observed correlations among variables as possible physiological status estimates in trout. Moreover, the obtained factors were tested for correlation with widely used indexes, the hepatosomatic and splenosomatic indexes.

## 2. Materials and Methods

This research was conducted on a serum chemistry variables dataset from a previous study, designed to assess normal blood chemistry ranges in rainbow trout. Accordingly, details on sampled fish, fish biometrics, and analytical techniques have been already described [3]. In brief:

### 2.1. Experimental Fish

Forty-five 24 h fasted rainbow trout (biometrics presented in Table 1) were randomly sampled from a local fish supplier during the same season (end April–middle June). Trout were housed in a raceway fed with well water at approximately constant physicochemical parameters throughout the year. Fish were sampled during regular slaughtering according to European Community regulations (Council Regulation (EC) N. 1099/2009). In particular, fish were stunned by a sharp blow to the head immediately after netting, paying attention to standardize fish sampling, in order to minimize handling stress and the possible occurrence of uncontrolled sources of variation in the tested blood variables. Fish were clinically healthy and underwent complete necropsy to exclude gross pathology and the presence of visible parasites. Moreover, evidence of tissue reaction or damage was ruled out by a microscopic exam of gill filaments, skin and intestinal scrapings, and liver and kidney imprints.

### 2.2. Blood Sampling and Analysis

Blood was collected by caudal venipuncture immediately after stunning, allowed to clot for approximately 10 min, then centrifuged for 10 min in a refrigerated centrifuge. Hemolyzed samples were discarded. Collected serum was analyzed, using commercially available kits (Olympus Systems Reagents, Olympus Life and Material Science Europe GmbH, Hamburg, Germany) and with an automated biochemical analyzer (Olympus AU400, Mishima Olympus Co. Ltd, Shizuoka, Japan), for the following variables: glucose (GLU), urea (blood urea nitrogen, (BUN)), creatinine (CREA), total bilirubin (TBIL), aspartate aminotransferase (EC 2.6.1.1) (AST), alanine aminotransferase (EC 2.6.1.2) (ALT), alkaline phosphatase (EC 3.1.3.1) (ALP), creatine phosphokinase (EC 2.7.3.2) (CPK), lactate dehydrogenase (EC 1.1.1.27) (LDH), total protein (TPRO), albumin (ALB), triglycerides (TRIG), cholesterol (CHOL), Ca, P, and Mg. An ion-selective electrode (ISE) unit on the same analyzer was used to assess serum Na, K, and Cl.

### 2.3. Statistical Analysis

Serum chemistry variables were preliminarily screened for possible extreme cases by means of a boxplot graph to identify and exclude from analysis cases with values of more than 3 box lengths (interquartile range) from the upper and lower edge of the box. Thirty-nine samples were analyzed further. The mean and standard error for each variable are reported in Table 2.

Exploratory factor analysis was performed on the resulting dataset, using generalized least squares as the estimation method. The aim of this multivariate method is to reduce data dimensionality and to identify relatively few underlying factors explaining the observed correlations among variables. Furthermore, a lower number of independent factors may be obtained starting from many correlated variables, and the former may be used for subsequent analysis, simplifying the analytical task [15,16]. In the present study, the aim was mainly the latter in order to reduce the number of analytical variables, to test for a possible correlation with traditionally widely used biometric indexes (Pearson and Spearman correlation methods), and to develop possible indexes of fish physiological status and general wellness to be adopted in confined, artificial (fish farming) and/or natural systems. SPSS 14.0.1 (SPSS Inc., Chicago, IL, USA) and JASP 0.14.1 [17] were used as statistical packages.

## 3. Results

According to the Kaiser–Meyer–Olkin test results, five serum chemistry variables (ALT, CPK, Ca, Na, and Cl) showed unacceptable sampling adequacy (lower than 0.50, according to Kaiser [18]) and were discarded from further analysis. The overall and relative measure of sampling adequacy is reported in Table 3. It should be stressed that sampling adequacy is a measure of the proportion of variance among variables that might be a common variance to evaluate how data are suited for factor analysis [18]. Therefore, this does not exclude per se the possible clinical diagnostic relevance of the discarded variables.

The remaining variables were screened for commonality to evaluate how the percentage of variance of factor was explained by each correlated variable. All the variables showed sufficient commonality.

**Figure 1:**
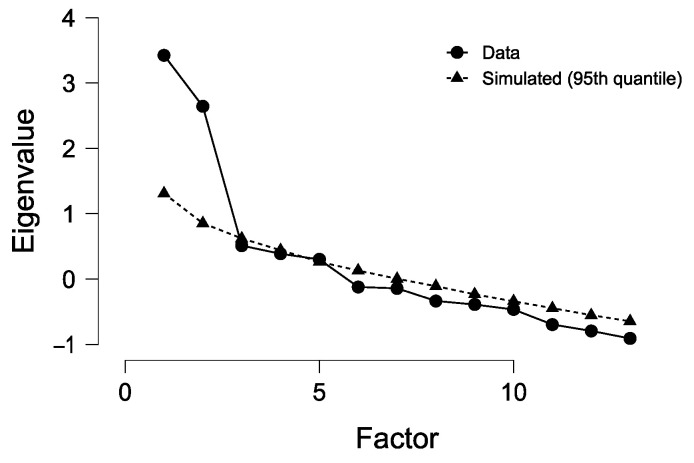
Screen plot. The plot provides information on how much variance in the data, in terms of eigenvalues, is explained by each factor.

Three factors were extracted by means of the generalized least squares extraction method, explaining at best the observed correlations among the analyzed serum chemistry variables (Figure 1).

The adequacy of the model was tested with the chi-square statistic. The null hypothesis and, as a consequence, the model were not rejected. The orthogonal rotation equamax ensured the best partition of variable correlations among factors. Extracted components and the related explained variance after rotation are reported in Table 4.

The rotated component matrix is reported in Table 5, where only factor loadings higher than or equal to 0.400 are reported for each extracted factor.

**Figure 2:**
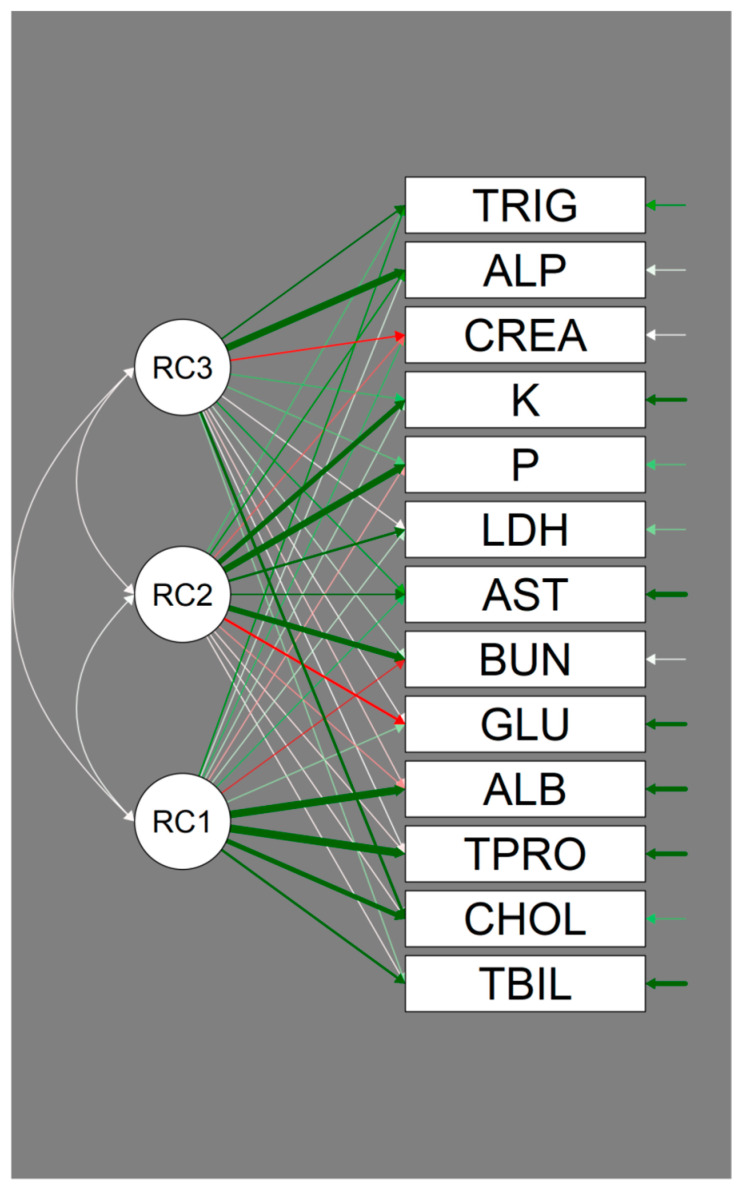
Path diagram. Factors are represented by circles and serum chemistry variables by boxes. The arrows going from the factors to the variables represent the loading from the factor on the variable, with green indicating a positive loading and red a negative loading. The wider the arrows, the higher the loading. Arrows below the set factor loading (0.400) are transparentized.

The path diagram shows graphically the loading from each factor on each variable (Figure 2).

Accordingly, TBIL, TPRO, ALB, and CHOL are clearly associated with Factor 1, and the latter result is also associated with Factor 3. GLU, BUN, AST, LDH, P, and K are associated with Factor 2, whereas CREA, ALP, and TRIG are mainly associated with Factor 3, with CHOL being shared with Factor 1, as underlined in Table 5.

The obtained factor scores (factor scores method: regression) correlate negatively with some of the biometric indexes. In particular, Factor 1 correlates with the hepatosomatic index (Spearman’s rho, −0.342; *p* < 0.05) and Factors 2 and 3 with the splenosomatic index (respectively, Spearman’s rho, −0.322 and −0.394; *p* < 0.05).

## 4. Discussion

All serum variables fell within the normal ranges for rainbow trout, as previously assessed [3].

No direct comparison in terms of confirmatory factor analysis can be made with previous studies because no author has previously applied exploratory factor analysis to a trout serum chemistry dataset. Nevertheless, Wagner et al. [19] performed factor analysis on six multivariate blood chemistry datasets in a related species, *Oncorhynchus tshawytscha* (Walbaum, 1792). In particular, four underlying factors were obtained: a “nutritional factor,” relying on TPRO, CHOL, Ca, and ALP; a “tissue damage factor,” relying on ALT, AST, and CPK; a “lipid metabolism factor,” relying on triacylglycerol lipase and TRIG; and a “stress factor,” relying on cortisol, GLU, Na, and Cl. In the present study, the dataset did not include cortisol and triacylglycerol lipase. Moreover, other substantial differences appeared with regard to the extracted factors (3 vs. 4 factors), the excluded variables (ALT, CPK, Na, and Cl), and the variable partition among factors.

Factor 1 relies, in order of decreasing strength, on TPRO, ALB, CHOL, and TBIL. Furthermore, TRIG should be considered, though their factor loading value of 0.385 was lower than the settled threshold. Albumin is a relatively small fraction of total blood protein in trout, though protein fractions have not been extensively studied compared to mammals [20]. Nevertheless, and apart from a relatively low presence of immunoglobulins (less than 10% of total protein in healthy fish), the vast majority of blood protein fractions are produced by the liver [1,3,20,21,22]. Moreover, blood proteins are transporters of cholesterol and lipids in general in the form of liver-synthesized lipoproteins [1,23,24,25,26,27]. Fish lack subcutaneous fat as significant lipid storage, with the liver and/or muscle being deputed to the latter task, according to the species. Very-low-density plasma lipoproteins are reported to reflect where lipids are stored, either in the liver or muscle [26]. With regard to bilirubin, unconjugated bilirubin, the predominant form in physiological conditions, is known to be ligated to albumin in mammals and with a high-density lipoprotein in *Oncorhynchus keta* (Walbaum, 1792) [1,28,29]. Accordingly, this reflects the relationship between TBIL and TPRO (they showed a strong linear correlation: Pearson’s r, 0.566; *p* < 0.01) and, ultimately, Factor 1.

The hepatosomatic index is an aspecific liver condition index. Given the central role of the liver in fish nutrients and toxin metabolism, it has been used in different studies to test for nutrition state adequacy, liver nutrient/toxin overload, liver nutrient depletion, etc. [13,30,31,32]. As a result, and given also the correlation of Factor 1 related scores with the hepatosomatic index, the author proposes to consider Factor 1 as a possible protein (liver) metabolism factor.

Factor 2 relies, in order of decreasing strength, on P, BUN, K, GLU, LDH, and AST. Alkaline phosphatase should also be considered, though its factor loading (0.393) was lower than the settled threshold. Phosphorus along with Ca are the most represented macroelements in vertebrates. Values ranging from 20,500 to 16,700 ppm of wet body mass are reported in Atlantic salmon, according to body mass (respectively, from <0.3 to <1500 g), whereas P content increases with the condition factor in rainbow trout [33,34]. Because of its enzymatic action, ALP is closely related to P metabolism, and its correlation was also observed in trout, though blood P was not considered an optimal indicator of P status in the latter species [35]. There is concern about P excretion in reared fish and its environmental effect, with particular regard to eutrophication [36,37]. Excess P may be released in the blood from the cell cytoplasm and membrane after cell injury, with particular regard to erythrocyte in the course of hemolysis [1].

Ammonia is the principal nitrogen waste in adult teleost fish, and gills are the main route of excretion [38,39]. Nevertheless, there are other nitrogen excretion forms, the most relevant of which is urea. In rainbow trout, ammonia nitrogen accounted for 53 to 68% of total nitrogen waste and urea nitrogen for 6 to 10% [38]. Urea is produced by uricolysis and/or arginolysis, though the presence of the ornithine–urea cycle enzymes have been observed in larval trout to account for impaired ammonia excretion caused by the absence of functional gills and the presence of an acellular chorion (egg capsule), limiting organism-to-blood diffusion [40]. Interestingly, chronic cortisol elevation was shown to increase BUN concentration and its branchial and renal excretion by threefold [41]. More recently, and interestingly, Clark et al. [42] reported that the genes related to the urea cycle and polyamine synthesis in rainbow trout show dynamic expression responses to inflammation as a possible route of arginine recycling.

Glucose is the most important biological monosaccharide, a privileged or unique energy source for many cell types [43]. The liver has a central role in glucose homeostasis through glucose synthesis and storage. The kidney should also be considered for its contribution to glucose synthesis along with muscle for its contribution to glucose use. Moreover, the intestine, as the first line in nutrient uptake, plays an important role in glucose homeostasis. In trout, similarly to other carnivorous fish, glucose metabolism is somewhat different from typical mammals, relying mainly on gluconeogenesis at hepatic, renal, and intestine levels rather than on food carbohydrate use and glycogenolysis [44,45]. One of the most important physiological causes of blood glucose rise in mammals is excitement and stress [1]. Trout showed a reduced stress response compared to other less domesticated fish (e.g., Eurasian perch (*Perca fluviatilis* Linnaeus, 1758)), particularly in repeatedly stressed exemplars [46]. Nevertheless, stress causes blood cortisol and glucose to increase, stressing the need to standardize the sampling strategy in order to mitigate its possible impact.

Aspartate aminotransferase is a cytoplasmic and mitochondrial enzyme, catalyzing the deamination of aspartate to form oxaloacetate. Lactic dehydrogenase is a cytoplasmic enzyme, catalyzing the conversion of pyruvate to lactate. The main cellular sources of serum AST and LDH activity are liver cells, skeletal muscle cells, cardiac muscle cells, and erythrocytes [1]. Aspartate aminotransferase and lactate dehydrogenase isozymes have been reported in brown trout (*Salmo trutta* Linnaeus, 1758), respectively, in the liver, muscle, and eye (AST) and in the liver, muscle, heart, kidney, eye (LDH) [47]. Lactate dehydrogenase polymorphism was reported in rainbow trout, with subunits in the liver and gill [48].

Cell damage is the major mechanism leading to increased AST and LDH serum activity. Regarding ALP, induction is considered the main cause of increased serum activity, according to species. In mammals, different ALP isoforms are known, from the liver, bone, intestine, mammary gland; therefore, the main cellular sources of serum ALP are liver cells, biliary epithelial cells, osteoblasts, and mammary gland cells. Bone, liver, and intestine isoforms are reported in rainbow trout [35,49]. In fish, another isoform is reported from the skin and epidermal mucus, with possible anti-inflammatory and innate immunity function, showing a correlation with serum ALP activity [50].

Cytosolic (e.g., ALT, LDH) and membrane enzymes (e.g., ALP) may be released in the blood by reversible or irreversible cell damage, comprising membrane blebbing, increased membrane permeation, or necrosis. Mitochondrial enzymes (e.g., AST) need more severe tissue damage to be released [1,51,52]. It should be stressed that serum enzyme activity in clinically healthy organisms is assumed to result from physiologic cellular turnover [1]. Moreover, the transitory elevation of the activity of some serum enzymes over normal ranges may result as a consequence of intense physical exercise, without any pathological consequence for the individual [53,54]. The effect of the temperature at which enzyme activity is analyzed (37 °C is the internationally accepted reference temperature) should be taken into account too, in particular when poikilothermic vertebrates, like fish, are studied [3].

Potassium, along with Na, is one of the most representative monovalent cations. Because of the Na/K pump action, it is mainly intracellular, though it flows extracellularly during cell membrane repolarization [43,55]. Moreover, its transmembrane movement is related to H^+^ movement, in the regulation of blood pH, and to cell volume [1,56,57]. Therefore, an increase in serum K concentration may be due to morphofunctional membrane alteration, cell lysis, in particular hemolysis, inorganic acidosis, and renal insufficiency or failure [1,56].

Based upon the aforementioned considerations, the author proposes to consider Factor 2 as a possible cell turnover factor.

Factor 3 relies, in order of factor loading, on ALP, CHOL, TRIG, and CREA. Very interestingly, in lymph ALP, an increase is reported in response to lipid feeding, possibly stimulated by the uptake and/or the re-esterification of lipid digestion products [58]. Moreover, its involvement in chylomicron formation and fatty acid metabolism has been suggested [59], and its possible involvement in cholesterol metabolism has been recently proposed [60], linking ALP with CHOL and TRIG. Plasma lipoproteins have been characterized in rainbow trout according to age, sex, and season. Referring to human clinical standards, trout may be considered hyperlipidemic and hyperlipoproteinemic; moreover, the apolipoprotein compositions, according to each lipoprotein class, and their similitude with human counterparts have been described [61]. In trout, high-density lipoproteins (HDLs) are catabolized mainly in the kidney, stressing the key role of the latter in the endocytosis of exogenous and endogenous macromolecules [62]. Conversely, native low-density lipoproteins (LDLs) are catabolized mainly in the liver, though modified LDLs are cleared by a scavenger receptor pathway in the kidney [63,64]. Creatinine derives from muscular creatine degradation and is cleared by glomerular filtration in the kidney and by the alimentary canal. Differently from urea, it is not resorbed by renal tubules, accounting for the glomerular filtration rate [1]. Because fish rely mainly on gills for excretion, there is a generalized lack of information about renal CREA clearance in fish [65]. Apart from altered clearance, CREA may increase as a consequence of muscle damage, including intense agonist activity [1]. Recently, a blood CREA increase has been related to muscle activity in trout [4]. Moreover, muscle, along with visceral adipose tissue and the liver, is also involved in lipid metabolism. In particular, trout, as a “fatty fish,” has a relatively low amount of hepatic lipid storage and, conversely, is able to store a significant level of lipid in its musculature to be used particularly during endurance swimming and to be mobilized during starvation [66,67].

Referring to previous considerations, Factor 3 can be considered as a lipid (muscle) metabolism factor.

With regard to the negative correlation between Factors 2 and 3 with the splenosomatic index, the latter has been used as a rough estimate of immunocompetence, with a higher value associated with immune activation [68]. Moreover, spleen mass is affected by acute stress. In particular, acute spleen mass reduction is observed either as a result of the massive release of erythrocytes into the bloodstream or as a result of leukocyte glucocorticoid-mediated activation, recruitment, and tissue redistribution [69,70,71,72]. The widely known lymphocyte glucocorticoid-induced apoptosis [73] should also be considered as a possible source of variation in spleen mass. Assuming the implication of physiological stress (eustress) in the modulation of Factor 2, a possible conflicting result emerges with regard to glucose, which contributes negatively to the loading of the previous factor and correlates the latter negatively with the splenosomatic index. Nevertheless, the immune system relies strongly on blood glucose [74], thus its availability may correspond to a rather competent immune system and consequently to an increased splenosomatic index. With regard to the negative factor loading of CREA, admitting its possible marker function of muscle activity, it may be related to an immunomodulating effect of exercise, as reported in humans, in which moderate and regular exercise is associated with immunostimulation, whereas strenuous exercise may cause temporary immunodepression [75]. With regard to lipids, their qualitative and/or quantitative variation has proven immunomodulating activity also in trout [76,77]. This aspect highlights the possible role of CHOL and TRIG in Factor 3, particularly the correlation of this latter with the splenosomatic index. As a consequence, both Factors 2 and 3 may account for trout immunocompetence, deserving nevertheless further research and definitive confirmation using specific and aspecific immunological markers and controlling for possible confounding factors able to affect spleen mass.

## 5. Conclusions

The exploratory factor analysis of 18 serum chemistry variables resulted in the selection of 13 highly correlated variables. Moreover, three underlying factors were identified that explained the observed correlations among variables, the scores of which correlated negatively with some traditionally used biometric indexes, such as the hepatosomatic (Factor 1) and splenosomatic indexes (Factors 2 and 3).

Because factor analysis is an exploratory analysis, the aim of which is to reduce data dimensionality and to identify relatively few underlying factors explaining the observed correlations among variables, factors have been tentatively associated with: protein (liver) metabolism (Factor 1), cell turnover (Factor 2), lipid (muscle) metabolism (Factor 3).

Further research is warranted for a definitive confirmation of the physiological significance and for the validation of the described underlying factors in order to adopt them in the assessment of trout physiological status and general wellness both in confined, artificial, and/or natural systems.

## Figures and Tables

**Table 1:** Mean and standard error of fish biometrics.

	Mean	Std. Error
Standard length (cm)	26.4	0.7
Body mass (g)	254.1	20.0
Condition factor	1.3	0.0
Liver mass (g)	2.8	0.2
Hepatosomatic index	1.2	0.1
Splenic mass (g)	0.6	0.1
Splenosomatic index	0.2	0.0

Condition factor (100 body mass standard length^−3^); hepatosomatic index (100 liver mass body mass^−1^); splenosomatic index (100 splenic mass body mass^−1^).

**Table 2:** Mean and standard error of serum chemistry variables.

Serum Chemistry Variables	Mean	Std. Error
Glucose (mg dL^−1^)	116.13	10.85
Blood urea nitrogen (mg dL^−1^)	4.08	0.21
Creatinine (mg dL^−1^)	0.30	0.01
Total bilirubin (mg dL^−1^)	0.04	0.00
Albumin (g dL^−1^)	1.37	0.05
Total protein (g dL^−1^)	3.55	0.14
Cholesterol (mg dL^−1^)	249.97	10.98
Triglycerides (mg dL^−1^)	343.38	24.35
Aspartate aminotransferase (U L^−1^)	435.67	25.92
Alanine aminotransferase (U L^−1^)	11.44	0.82
Alkaline phosphatase (U L^−1^)	179.59	20.56
Creatine phosphokinase (U L^−1^)	1148.64	154.90
Lactate dehydrogenase (U L^−1^)	2414.31	138.03
Ca (mg dL^−1^)	12.32	0.20
P (mg dL^−1^)	21.55	1.24
Na (mEq L^−1^)	154.51	0.83
K (mEq L^−1^)	4.24	0.52
Cl (mEq L^−1^)	129.38	0.80

**Table 3:** Kaiser–Meyer–Olkin test results.

	MSA ^a^
Overall MSA	0.667
Glucose	0.593
Blood urea nitrogen	0.706
Creatinine	0.550
Total bilirubin	0.576
Aspartate aminotransferase	0.702
Alkaline phosphatase	0.556
Lactate dehydrogenase	0.545
P	0.653
Cholesterol	0.785
Triglycerides	0.760
Total protein	0.675
Albumin	0.674
K	0.850

^a^ Measure of sampling adequacy.

**Table 4:** Factor characteristics ^a^.

	Sum of Squared Loadings	Proportion Variance	Cumulative Variance
Factor 1	3.143	0.242	0.242
Factor 2	2.936	0.226	0.468
Factor 3	1.699	0.131	0.598

^a^ The applied rotation method is equamax.

**Table 5:** Factor loadings (structure matrix) ^a,b^.

	Factor 1	Factor 2	Factor 3
Glucose		−0.508	
Blood urea nitrogen		0.799	
Creatinine			−0.416
Total bilirubin	0.498		
Aspartate aminotransferase		0.403	
Alkaline phosphatase			0.910
Lactate dehydrogenase		0.496	
P		0.880	
Cholesterol	**0.714**		**0.527**
Triglycerides			0.422
Total protein	0.999		
Albumin	0.966		
K		0.745	

^a^ The applied rotation method is equamax. ^b^ Factor loadings higher than or equal to 0.400 and shared by different factors are bolded.
